# Male partner antenatal attendance and HIV testing in eastern Uganda: a randomized facility-based intervention trial

**DOI:** 10.1186/1758-2652-14-43

**Published:** 2011-09-13

**Authors:** Robert Byamugisha, Anne N Åstrøm, Grace Ndeezi, Charles AS Karamagi, Thorkild Tylleskär, James K Tumwine

**Affiliations:** 1 Department of Obstetrics and Gynaecology, Mbale Regional Referral Hospital, PO Box 921, Mbale, Uganda; 2 Department of Clinical Dentistry, Faculty of Medicine and Dentistry, University of Bergen, Postbox 7804, N-5020 Bergen, Norway; 3 Department of Paediatrics and Child Health, School of Medicine, Makerere University College of Health Sciences, PO Box 7072, Kampala, Uganda; 4 Clinical Epidemiology unit, School of Medicine, Makerere University College of Health Sciences, PO Box 7072, Kampala, Uganda; 5 Centre for International Health, Faculty of Medicine and Dentistry, University of Bergen, Postbox 7804, N-5020 Bergen, Norway

## Abstract

**Background:**

The objective of the study was to evaluate the effect of a written invitation letter to the spouses of new antenatal clinic attendees on attendance by couples and on male partner acceptance of HIV testing at subsequent antenatal clinic visits.

**Methods:**

The trial was conducted with 1060 new attendees from October 2009 to February 2010 in an antenatal clinic at Mbale Regional Referral Hospital, Mbale District, eastern Uganda. The intervention comprised an invitation letter delivered to the spouses of new antenatal attendees, while the control group received an information letter, a leaflet, concerning antenatal care. The primary outcome measure was the proportion of pregnant women who attended antenatal care with their male partners during a follow-up period of four weeks. Eligible pregnant women were randomly assigned to the intervention or non-intervention groups using a randomization sequence, which was computer generated utilizing a random sequence generator (RANDOM ORG) that employed a simple randomization procedure. Respondents, health workers and research assistants were masked to group assignments.

**Results:**

The trial was completed with 530 women enrolled in each group. Participants were analyzed as originally assigned (intention to treat). For the primary outcome, the percentage of trial participants who attended the antenatal clinic with their partners were 16.2% (86/530) and 14.2% (75/530) in the intervention and non-intervention groups, respectively (OR = 1.2; 95% CI: 0.8, 1.6). For the secondary outcome, most of the 161 male partners attended the antenatal clinic; 82 of 86 (95%) in the intervention group and 68 of 75 (91%) in the non-intervention group were tested for HIV (OR = 2.1; 95% CI: 0.6 to 7.5).

**Conclusions:**

The effect of the intervention and the control on couple antenatal attendance was similar. In addition, the trial demonstrated that a simple intervention, such as a letter to the spouse, could increase couple antenatal clinic attendance by 10%. Significantly, the majority of male partners who attended the antenatal clinic accepted HIV testing. Therefore, to further evaluate this simple and cost-effective intervention method, adequately powered studies are required to assess its effectiveness in increasing partner participation in antenatal clinics and the programme for prevention of mother to child transmission of HIV.

**Trial Registration:**

**ClinicalTrials.gov Identifier**: NCT01144234.

## Background

Approximately 370,000 children were newly infected with HIV during 2009 through mother to child transmission [1]. Sub-Saharan Africa, the region most affected by HIV, accounts for 67% of HIV infections worldwide and 91% of new infections among children [2]. HIV counselling and testing is the access point to HIV prevention, care and treatment programmes. However, access to services for preventing mother to child transmission of HIV in low- and middle-income countries remains limited, with only 26% of pregnant women in such countries receiving HIV tests during 2009 [1].

Following World Health Organization (WHO) recommendations [3], routine antenatal counselling and testing for HIV has been introduced into prevention of mother to child transmission (PMTCT) of HIV programmes in resource-limited settings; this has increased HIV testing rates among antenatal attendees in several sub-Saharan countries [4-10]. Engaging men as partners is a critical component of the PMTCT programme, but their involvement in antenatal care (ANC) and PMTCT services has remained low [11-15]. However, men exercise a huge influence on their wives regarding sexual and reproductive health issues [16,17]. Male involvement in antenatal HIV counselling and testing increases the use of PMTCT interventions in resource-limited settings [18-21] and is associated with reduced mother to child transmission of HIV-1 and reduced infant mortality [22].

In Uganda, HIV is a major public health problem and there was an estimated adult HIV prevalence rate of 6.7% in 2006 [23], yet only 15% of adults know their HIV status. The PMTCT programme was launched in Uganda in 2001 and is currently integrated into mainstream antenatal care services. However, the proportion of male partners of pregnant women tested in antenatal clinics for HIV is low. Further, the proportion of HIV discordance among couples who test is high, ranging from 35% to 50% [23-26]. One objective of the Uganda's policy change from antenatal voluntary counselling and testing (VCT) to routine antenatal counselling and testing in June 2006 was to increase the proportion of male partners of pregnant women offered HIV counselling and testing services from the PMTCT programme from 3% to 25% by 2010 [27].

A study in Mbale Regional Referral Hospital in Mbale District, eastern Uganda, revealed high antenatal HIV testing rates (more than 90%) among pregnant women [5] as a result of routine antenatal counselling and testing. Nonetheless, antenatal attendance and HIV testing among their male partners remained very low (4.7%) [28] despite the fact that counsellors encouraged the antenatal attendees to invite their male partners for clinic attendance and HIV testing [29]. However, measures to increase male partner participation in PMTCT programmes in Uganda have not been investigated. Therefore, this trial was conducted to evaluate the effect of a written invitation letter delivered to the spouses of women attending their first antenatal visit on couple attendance and partner acceptance of HIV testing at subsequent antenatal clinic appointments within a four-week follow-up period.

## Methods

A randomized, parallel group, health facility-based intervention trial was conducted among 1060 new attendees (530 individuals in the intervention group and 530 in the control group) at the antenatal clinic in Mbale Regional Referral Hospital from October 2009 to February 2010. The trial setting has been described elsewhere [29]. Routine HIV counselling and testing were carried out according to the Uganda Ministry of Health guidelines (2006) [27].

A sequential HIV testing algorithm with same-day results, which includes three rapid tests, is carried out using one blood sample: *Determine HIV 1 ⁄ 2 assay (Abbott Laboratories, Abbott Park, IL, USA) *for first screening; *STAT-PAK HIV 1 ⁄ 2 dipstick assay (Chembio Diagnostic Systems Inc.) *as a second test; and *Uni-Gold Recombigen HIV (Trinity Biotech, Wicklow, Ireland) *as a "tie-breaker". An ANC attendee is classified as uninfected if Determine is negative and as HIV infected if the *Determine *and *STAT-PAK *tests are positive. Discordant *Determine *and *STAT-PAK *blood samples are further tested using the *Uni-Gold *test. The HIV test result is reported as positive if the *Uni-Gold *test is positive and as negative if the *STAT-PAK *and *Uni-Gold *tests are negative.

Since 2006, ANC clinic attendees who are HIV-positive undergo CD4 cell counts before being administered appropriate treatments according to the national PMTCT guidelines [27]. The sample size for this trial was calculated using a computer programme, OpenEpi, version 2 (http://www.openepi.com/SampleSize/SSCohort.htm). Based on the assumptions that antenatal couple attendance and HIV testing would increase from 4.5% (without intervention) to 9% (with intervention) with 80% power and 95% confidence intervals, 1060 new antenatal attendees, 530 in each group, were enrolled into the trial. The trial was completed four weeks after enrolment of the last participant.

Eligible trial participants were new attendees, aged 15 years or above, who agreed to attend subsequent antenatal visit(s) within the four-week follow-up period at Mbale Hospital, and were willing to give the invitation and information letters to their male partners. A male partner was defined in this trial as the male who impregnated the antenatal attendee in the current pregnancy. The exclusion criteria included women who attended with their spouses at the first antenatal visit or did not consent to participate in the trial, or had spouses who were inaccessible.

Women attending without spouses at the first antenatal visit were identified at reception in the antenatal clinic. They were tracked until they had undergone all the standard clinic procedures (namely registration, health education, pre- and post-test counselling for HIV, HIV testing, obstetric examination and treatment). Individuals were approached by research assistants and informed about the trial's objective and the intervention. Those who agreed to participate in the trial by providing written consent were enrolled into the trial using an enrolment form with a randomly generated identification number.

Each woman was provided with a letter addressed to her spouse and given an appointment for a return visit two weeks later. If the participant was not able to attend with her partner on the scheduled visit, she was given another appointment for a return visit two weeks later. Their identification numbers were marked on their antenatal cards to aid follow up at the next clinic visit. The importance of adhering to their ANC visits was uniformly emphasized. Women who did not return to the antenatal clinic for their scheduled visits during the four-week follow-up period were classified as "lost to follow up".

At enrolment into the trial by the research assistants, participants were randomly assigned to two parallel groups, the intervention and non-intervention groups, with an allocation ratio of 1:1. The intervention comprised an invitation letter addressed to the male partner of the woman attending her first ANC visit, requesting him to accompany her on the next ANC visit. The comparative arm (non-intervention group) received a letter containing information concerning services offered in the antenatal clinic at Mbale Regional Referral Hospital.

Detailed in the invitation letter was the following information: the appointment date of the woman's next antenatal visit; that the antenatal and PMTCT services are free (no user charges); that these services are beneficial to the couple and their unborn baby, and that their utilization by men is low; that he was cordially invited to accompany the woman at her next scheduled antenatal visit to discuss important issues concerning her antenatal care; and that the time spent in hospital would be minimal.

The information letter, the leaflet, contained details concerning services provided in the antenatal clinic, and these included checking the woman's blood pressure to detect and manage high blood pressure during pregnancy. It explained that the woman's urine is tested for protein, sugar and infections, that her blood is checked for low haemoglobin levels, and that her abdomen is examined to investigate the wellbeing of the baby. Lastly, the leaflet informed the woman and her partner that PMTCT services in the clinic were free of charge. The letters were of similar length, and content was comparable, with one being an invitation and the other being an information leaflet only. Each letter was duly signed by the principal investigator.

The random allocation list of the identification numbers was randomly generated by an independent statistician from TASO Uganda (The AIDS Support Organisation). A random sequence generator (computer programme) at the RANDOM.ORG website [30], which employed a simple randomization procedure, was utilized. The random numbers were hand-written at the bottom of the back page of the letters for the intervention and non-intervention groups by the principal investigator. Each letter was inserted into an opaque envelope and sealed with adhesive glue to ensure that participants would not open their husbands' letters.

The corresponding randomization number was written on the back of the envelope. No antenatal clinic staff, co-investigators, research assistants or pregnant women knew whether the sealed envelopes contained the intervention or non-intervention letters. The randomization code was kept securely by the principal investigator. Each woman enrolled into the trial by the research assistants was given an identification number (serial number). The appropriate envelope, whose randomization number corresponded to the serial number, was selected and given to the participant to give to her spouse. The randomization code was revealed to the co-investigators during data analysis.

Data were collected by five trained research assistants using a standardized, pre-tested questionnaire, administered to participants in English or Lumasaba (local language) during exit interviews at subsequent clinic visits during the four-week follow-up period. Four weeks after enrolment into the trial, women who had not returned for their subsequent antenatal visits were deemed to have been lost to follow up. The research assistants were knowledgeable in the local language and interview techniques, and had been trained in terms of the trial objectives and methods.

The structured interview covered topics concerning the participant's education, occupation, religion, ethnic group, number of pregnancies, household assets, opinions and experiences relating to routine HIV counselling and HIV testing in the antenatal clinic, and knowledge of mother to child transmission of HIV and infant feeding options for HIV-infected mothers. Furthermore, her partner's age, occupation and education was discussed. Participants were asked about partner clinic attendance and partner antenatal HIV testing acceptance. Questionnaires were checked for completeness at the end of each day by the principal investigator and clarification sought from research assistants when queries arose. Data were entered using EpiData version 3.1 [31] by two data entry clerks and were validated by the principal investigator. The data file was exported to PASW Statistics 18 [32] (formerly SPSS) for analysis.

Ethical clearance to conduct the trial was obtained from the Research and Ethics Committee of the School of Medicine, Makerere University, and from the Uganda National Council of Science and Technology. Permission to conduct the trial in the antenatal clinic was obtained from the Mbale Regional Referral Hospital administration through the local institutional review board. Written informed consent was provided by all trial participants. The trial was registered with the ClinicalTrials.gov registry (Identifier: NCT01144234).

The pre-specified primary outcome measure of the trial was the proportion of pregnant women who attended ANC with their partners at the subsequent antenatal visit. The secondary outcome measure was the proportion of men who accepted routine antenatal HIV testing. All participants were included in the analysis for the primary outcome measure in the groups to which they were originally assigned (intention to treat); analysis for the secondary outcome included only participants who attended their scheduled return clinic visits during the follow-up period (per protocol analysis).

The socio-demographic characteristics of the trial participants in the intervention and non-intervention groups were compared using independent sample *t*-test for continuous variables and the Pearson chi-square test for categorical variables. Correlates of couple ANC attendance (male antenatal clinic attendance) and male partner antenatal HIV-testing in the intervention and non-intervention groups was determined using the Pearson Chi-square test and the independent *t*-test. Multi-collinearity among the independent variables and outliers were investigated. Interactions were explored and binary logistic regression was used to test for confounding variables.

All variables that were significant at the level of p < 0.2 in binary analysis, and the age of participants, were retained in the multivariate regression model. All p values were two-tailed at a significance level of 5%. As indicators of model appropriateness, the goodness-of-fit test (Omnibus Tests of Model Coefficients) of each the final models for male partner antenatal attendance and for partner antenatal HIV-testing in the trial groups was significant (p < 0.05), and the Hosmer and Lemeshow goodness-of-fit test was not significant (p value > 0.05) (see tables 1 and 2).

**Table 1:** Correlates of couple antenatal attendance among 600 pregnant women at Mbale Regional Referral Hospital

Study participants' characteristics (Variables)^a^	Male partner antenatal clinic attendance in intervention group (N = 290)^b^				Male partner antenatal clinic attendance in non-intervention group (N = 310)^c^			
		
	Attended n (%)	Did not attend n (%)	Unadjusted OR^d ^(95% CI)	Adjusted OR (95% CI^e^)	Attended n (%)	Did not attend n (%)	Unadjusted OR (95% CI)	Adjusted OR (95% CI
*Age (years)*								
15-24	42 (28)	111 (72)	1	1	40 (24)	130 (77)	1	1
25 or more	44 (32)	93 (68)	1.3 (0.8-2.1)	1.2 (0.7-2.1)	30 (25)	105 (75)	1.1 (0.6-1.8)	1.0 (0.6-1.8)
*Education level*								
No or incomplete primary	34 (28)	86 (72)	1		28 (24)	88 (76)	1	
Completed primary	52 (31)	118 (69)	1.1 (0.7-1.9)		47 (24)	147 (76)	1.0 (0.6-1.7)	
*Occupation*								
Not salaried	47 (30)	109 (70)	1		59 (22)	207 (78)	1	1
Salaried	39 (29)	95 (71)	1.0 (0.6-1.6)		16 (36)	28 (64)	2.0 (1.0-4.0)	1.5 (0.7-3.2)
*Ethnic group*								
Bagisu	55 (30)	128 (70)	1		41 (21)	151 (79)	1	1
Non-Bagisu	31 (29)	76 (71)	0.9 (0.6-1.6)		34 (29)	84 (71)	1.5 (0.9-2.5)	1.6 (0.9-2.9)
*Religion*								
Muslim	30 (24)	93 (76)	1	1	29 (23)	97 (77)	1	
Christian	56 (34)	111 (67)	1.6 (0.9-2.6)	1.6 (0.9-2.6)	46 (25)	138 (75)	1.1 (0.7-1.9)	
*Asked partner permission to test for HIV*								
No	27 (22)	95 (78)	1	1	24 (17)	119 (83)	1	1
Yes	59 (35)	109 (65)	1.9 (1.1-3.2)^f^	1.9 (1.1-3.3)^f^	51 (31)	116 (69)	2.2 (1.3-3.8)^g^	1.8 (1.0-3.2)^f^
*Partner's age (years)*								
19-29	28 (29)	68 (71)	1		27 (26)	76 (74)	1	
30 or more	49 (37)	82 (63)	1.5 (0.8-2.6)		32 (26)	92 (74)	1.0 (0.3-1.8)	
*Partner's occupation*								
Not salaried	47 (30)	109 (70)	1		34 (20)	134 (80)	1	1
Salaried	39 (29)	95 (71)	1.0 (0.6-1.6)		41 (29)	101 (71)	1.6 (1.0-2.7)	1.4 (0.8-2.5)
*Partner's education level*								

No or incomplete primary	16 (27)	43 (73)	1		9 (16)	47 (84)	1	1
Completed primary	62 (32)	133 (68)	1.3 (0.7-2.4)		59 (29)	147 (71)	2.1 (1.0-4.5)	1.7 (0.8-3.8)

^a^Other variables not statistically significant in univariate analysis were: Participant's place of residence, marital status and total number of pregnancies. Age as a possible confounder and all variables that were significant at the level of p < 0.2 in univariate analysis were retained in the multivariate regression model. Multicollinearity and interaction among the independent variables, and outliers were checked for.

^b^The goodness-of-fit test (Omnibus tests of Coefficients) of the final logistic regression model in the intervention group was significant [Chi-square statistic (χ^2^) = 9.376, degrees of freedom (df) = 3, p = 0.025] and Hosmer and Lemeshow goodness of fit test was not significant [χ^2 ^= 2.785, df = 6, p = 0.835] as indicators of model appropriateness.

^c^For the non-intervention group, the goodness-of-fit test (Omnibus tests of Coefficients) of the final logistic regression model was significant [χ^2 ^= 13.018, df = 6, *p *= 0.043] and Hosmer and Lemeshow goodness of fit test was not significant [χ^2 ^= 5.617, df = 8, p = 0.690 as indicators of model appropriateness.

^d^OR: odds ratio

^e^CI: confidence interval

^f^Statistically significant: p < 0.05 (two-tailed)

^g^Statistically significant: p < 0.01(two-tailed)

**Table 2:** Correlates of male partner HIV testing in the antenatal clinic at Mbale Regional Referral Hospital, eastern Uganda

Study participants' characteristics (variables)^a^	Male HIV testing in antenatal clinic in intervention group (N = 290)^b^				Male HIV testing in antenatal clinic in non-intervention group (N = 310)^c^			
		
	Tested for HIV n (%)	Not tested for HIV n (%)	Unadjusted OR^d ^(95% CI)	Adjusted OR (95% CI^e^)	Tested for HIV n (%)	Not tested for HIV n (%)	Unadjusted OR (95% CI)	Adjusted OR (95% CI)
*Age (Years)*								
5-24	40 (26)	113 (74)	1	1	33 (19)	137 (81)	1	1
25 or more	42 (31)	95 (69)	1.2 (0.7-2.1)	1.2 (0.7-2.1)	35 (25)	105 (75)	1.4 (0.8-2.4)	1.3 (0.7-2.3)
*Education level *								
No or Incomplete primary	33 (28)	87 (72)	1		23 (20)	93 (80)	1	
Completed Primary	49 (29)	121 (71)	1.0 (0.9-1.2)		45 (23)	149 (77)	1.2 (0.7-2.1)	
*Occupation*								
Not salaried	72 (28)	188 (72)	1		53 (20)	213 (80)	1	1
Salaried	10 (33)	20 (67)	1.3 (0.6-2.9)		15 (34)	29 (66)	2.1 (1.0-4.2)	1.4 (0.6-3.1)
*Ethnic group*								
Bagisu	52 (28)	131 (72)	1		37 (19)	155 (81)	1	1
Non-Bagisu	30 (28)	77 (72)	1.0 (0.6-1.7)		31 (26)	87 (74)	1.5 (0.9-2.6)	1.6 (0.9-2.9)
*Religion*								
Muslim	27 (22)	96 (78)	1	1	25 (20)	101 (80)	1	
Christian	55 (33)	112 (67)	1.7 (1.0-3.0)^f^	1.7 (1.0-3.0)^f^	43 (23)	141 (77)	1.2 (0.7-2.1)	
*Asked partner permission to test for HIV*								
No	25 (21)	97 (79)	1	1	21 (15)	122 (85)	1	1
Yes	57 (34)	111 (66)	2.0 (1.2-3.4)^g^	2.0 (1.2-3.5)^f^	47 (28)	120 (72)	2.3 (1.3-4.0)^g^	1.9 (1.0-3.6)^f^
*Partner's age (years)*								
19-29	26 (27)	70 (73)	1		22 (21)	81 (79)	1	
30 or more	47 (36)	84 (64)	1.5 (0.8-2.7)		31 (25)	93 (75)	1.2 (0.7-2.3)	
*Partner's occupation*								
Not salaried	45 (29)	111 (71)	1		28 (17)	140 (83)	1	1
Salaried	37 (28)	97 (72)	0.9 (0.6-1.6)		40 (28)	102 (72)	2.0 (1.1-3.4)^f^	1.8 (1.0-3.3)^f^
*Partner's education level*								
No or incomplete primary	16 (27)	43 (73)	1		9 (16)	47 (84)	1	1
Completed primary	58 (30)	137 (70)	1.1 (0.6-2.2)		53 (26)	153 (74)	1.8 (0.8-3.9)	1.5 (0.7-3.4)

^a^Other variables not significant in univariate analysis were: participant's place of residence, marital status, and total number of pregnancies. Age as a possible confounder and all variables that were significant at the level of p < 0.2 in univariate analysis were retained in the multivariate regression model.

Multicollinearity and interaction among the independent variables, and outliers were checked for.

^b^The goodness-of-fit test (Omnibus tests of Coefficients) of the final logistic regression model in the intervention group was significant [chi-square statistic (χ^2^) = 11.362, degrees of freedom (df) = 3, p = 0.010] and Hosmer and Lemeshow goodness-of-fit test was not significant [χ^2 ^= 3.585, df = 6, p = 0.733] as indicators of model appropriateness.

^c^For the non-intervention group, the goodness-of-fit test (Omnibus tests of Coefficients) of the final logistic regression model was significant [χ^2 ^= 15.412, df = 6, p = 0.017] and Hosmer and Lemeshow goodness-of-fit test was not significant [χ^2 ^= 8.774, df = 8, p = 0.362 as indicators of model appropriateness.

^d^OR: odds ratio

^e^CI: confidence interval

^f^Statistically significant: p < 0.05 (two-tailed)

^g^Statistically significant: p < 0.01 (two-tailed)

## Results

### Trial population and follow up

**Figure 1:**
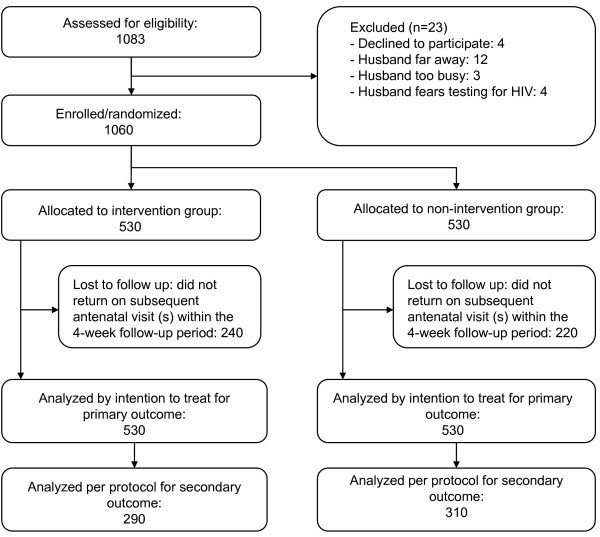
**Trial profile**.

A total of 1060 new antenatal attendees were enrolled and randomly assigned to the intervention and non-intervention groups (530 women in each group) (Figure 1). Of these, 290 and 310 pregnant women in the intervention and non-intervention groups, respectively, attended the subsequent two antenatal visits as scheduled; response rates were 55% (290/530) for the intervention group and 58% (310/530) for the non-intervention group. No major differences in the socio-demographic characteristics of the trial participants were recorded between the groups (Table 3).

**Table 3:** Demographic characteristics of study participants compared between intervention (N = 290) and non-intervention groups (N = 310)

Characteristics	Study groups		P value
		
	Intervention n (%)	Non-intervention n (%)	
*Age in years^a ^*			
15-24	153 (52.8)	170 (54.8)	0.64
25 or more	137 (47.2)	140 (45.2)	
*Place of residence *			
Rural	197 (67.9)	207 (66.8)	0.83
Urban	93 (32.1)	103 (33.2)	
*Number of pregnancies *			
One	64 (22.1)	72 (23.2)	0.48
Two or more	226 (77.9)	238 (76.8)	
*Education level *			
No education/incomplete primary	120 (41.4)	116 (37.4	0.43
Completed primary or more	170 (58.6)	194 (62.6)	
*Marital status *			
Single/divorced/widowed	4 (1.4)	6 (1.9)	0.75
Married/cohabiting	286 (98.6)	304 (98.1)	
*Occupation *			
Salaried	30 (10.3)	44 (14.2)	0.19
Not salaried	260 (89.7)	266 (85.8)	
*Ethnic group *			
Bagisu	183 (63.1)	192 (61.9)	0.83
Non-Bagisu	107 (36.9)	118 (38.1)	
*Religion *			
Muslim	123 (42.4)	126 (40.6)	0.72
Christian	167 (57.6)	184 (59.4)	
*Partner's age in years^b ^*			
19-29	96 (42.3)	103 (45.4)	0.51
30 or more	131 (57.7)	124 (54.6)	
*Partner's education level *			
No education/incomplete primary	59 (23.2)	56 (21.4)	0.45
Completed primary or more	195 (76.8)	206 (78.6)	
*Partner's occupation *			
Not salaried	156 (53.8)	168 (54.2)	0.99
Salaried	134 (46.2)	142 (45.8)	

^a^The median age of the pregnant women was 24 years in both the intervention [interquartile range (IQR): 20-28 years] and non-intervention (IQR: 21-29 years) groups.

^b^The male partners' median age was 30 years in the intervention group (IQR: 26-38 years) and non-intervention group (IQR: 26-35 years), respectively.

Analysis by intention to treat demonstrated that the proportions of participants who attended with their partners were 16.2% (86/530) and 14.2% (75/530) in the intervention and non-intervention groups, respectively (Odds Ratio, OR = 1.2; 95% Confidence Interval, CI: 0.8 to 1.6) (see Table 4). There was no difference between the intervention and control groups with respect to the main outcome variable using bivariate analysis [Pearson chi-square value (χ^2^) = 0.35, *p*-value = 0.55]. The majority of male partners in the intervention group (95%) and non-intervention group (91%), who attended the antenatal clinic with their spouses, accepted HIV testing. There was no statistically significant difference between the intervention and non-intervention groups with respect to male partner antenatal HIV testing using bivariate analysis (OR = 2.1; 95% CI: 0.6 to 7.5) and multivariate analysis (OR = 1.6; 95% CI: 0.4 to 6.8), table 4. The men in the two groups were similar (*p*-value = 0.39).

**Table 4:** Primary and secondary outcomes of the facility based-intervention study at Mbale Regional Referral Hospital, eastern Uganda

	Intervention group: n/N (%)	Non-intervention group: n/N (%)	Unadjusted **OR**^a ^**(95% CI**^b^**)**	Adjusted OR (95% CI)
**Primary outcome**				
Couple antenatal attendance				
*Intention to treat analysis*	86/530 (16.2)	75/530 (14.2)	1.2 (0.8-1.6)	
*Per protocol analysis*	86/290 (29.7)	75/310 (24.2)	1.3 (0.9-1.9)	1.5 (1.0-2.3)^c^
**Secondary outcome(s)**				
Partner accepted HIV test^d^	82/86 (95.3)	68/75 (90.7)	2.1 (0.6-7.5)	1.6 (0.4-6.8)^e^
Partner's HIV test results^f^				
HIV positive	3/82 (3.7)	0 (0)		
HIV negative	79/82 (96.3)	68/68 (100)		
Loss to follow up	240/530 (45.3)	220/530 (41.5)	1.2 (0.9-1.5)	
Participant's HIV test results				
HIV positive	11/290 (3.8)	14/310 (4.5)	0.8 (0.4-1.7)	
HIV negative	279/290 (96.2)	296/310 (95.5)	1	

^a^Odds ratio

^b^Confidence interval

^c^Adjusted for the participant's and male partner's age, occupation and education level, the couple antenatal attendance odds ratio

^d^Partner acceptance of antenatal HIV testing analyzed per protocol

^e^Adjusted for male partner's age, occupation and education level

^f^Fisher Exact test two-sided p value was 0.32

The majority of male partners (93%, 150 out of 161) in both trial arms who accepted antenatal HIV counselling and testing were HIV sero-negative. Three men in the intervention group tested positive for HIV (Table 4).

All the female antenatal attendees who participated in the trial accepted antenatal HIV testing. Twenty-five (11 in the intervention group and 14 in the control group) tested positive for HIV (Table 4). Significantly, the male partners of most of these HIV-positive women (84%; 21/25) were not tested for HIV in the antenatal clinic (10 men did not attend the clinic; 11 men attended, but declined to be tested). Of the five couples with known HIV test results, three were discordant: in one couple, the woman was HIV negative and male partner was HIV positive; in two couples, the women were HIV positive and the partners were HIV negative. Two couples were concordant (both partners had HIV-positive test results). Therefore, in this trial, of the 150 couples who accepted antenatal HIV testing, 2% (three of 150) were identified as HIV sero-discordant.

### Correlates of couple antenatal attendance and male partner antenatal HIV testing

Using multivariate logistic regression analysis, participants having asked for their partner's permission to test for HIV was the only variable significantly associated with couple antenatal attendance in the intervention group [adjusted OR (AOR) = 1.9; 95% CI: 1.1 to 3.3] and the comparative group (AOR = 1.8; 95% CI: 1.0 to 3.2) (see Table 1). The likelihood of partner antenatal attendance increased if the partner had completed primary school education (AOR = 1.7; 95% CI: 0.8 to 3.8), but this association was not statistically significant.

The trial demonstrated that the likelihood of male partner HIV testing increased if the participant had asked their partner for permission to test for HIV, and this was the case for the intervention (AOR = 2.0; 95% CI: 1.2 to 3.5) and non-intervention groups (AOR = 1.9; 95% CI: 1.0 to 3.6). In addition, the participant being a Christian (AOR = 1.7; 95% CI: 1.0 to 3.0) and their partner being salaried (AOR = 1.8; 95% CI: 1.0 to 3.3) were significantly associated with male partner acceptance of antenatal HIV testing (Table 2).

## Discussion

As far as we are aware, this is the second randomized clinical trial to evaluate the effects of a written invitation letter to spouses of antenatal attendees on partner antenatal clinic attendance in sub-Saharan Africa. The effect of the intervention (invitation letter) and the control (information leaflet) on couple antenatal attendance in the trial was similar. The invitation letter and the information leaflet increased couple attendance at the antenatal clinic from approximately 5% [28] to 16% and 14%, respectively. A simple intervention letter to the spouse could increase couple attendance by 10%. This cost-effective intervention could be implemented in almost all African ANC clinics with PMTCT.

The surprisingly equal effect in both arms of the trial could be because the invitation letter (intervention) and the information letter (control) had an official connotation and were perceived by the male partners to be credible as they originated from hospital. Therefore, these letters influenced male antenatal attendance decisions in similar ways, irrespective of the detailed content.

A recent study, carried out in northern Uganda, has documented that the likelihood of male partner antenatal attendance was increased if men were knowledgeable about antenatal care services and if they obtained health information from health workers [33]. The lack of any significant difference between the intervention and the control letter on couple antenatal attendance could be explained by the low power of the trial as a result of the high loss to follow up of trial participants.

The level of male antenatal attendance in this trial is higher than one carried out in northern Tanzania [11], but lower than those documented in studies from northern Uganda [33], central Kenya [22] and Khayelitsha, South Africa [34]. The age groups of the men in these studies were comparable with those of the male partners in the current trial. It was also reported in the northern Uganda study that the likelihood of male antenatal attendance was higher if men had attained secondary or higher level education [33], but partner education level was not significantly associated with male antenatal attendance in the current trial. The level of partner attendance in this trial was similar to that reported in a study in Nairobi, Kenya [35].

Significantly, the current trial demonstrated that the majority (more than 90%) of male partners who attended the antenatal clinic accepted HIV counselling and testing for HIV. A similar finding has been reported in other studies in the region [22,35]. The implication of this finding is that increasing male antenatal clinic attendance is vital for involving spouses of antenatal attendees in the PMTCT programme. A woman having sought a partner's permission for HIV testing was significantly associated with partner antenatal attendance and HIV testing, as demonstrated using multivariate analysis. A similar finding was reported in the Nairobi antenatal clinic study [35]. This suggests that improved communication between couples regarding HIV is an important factor in increasing the number of men accompanying their spouses to antenatal clinics and accessing HIV counselling and testing services.

However, there was a differential effect because the HIV sero-status of approximately 80% of the HIV-positive women's partners remained unknown, which constitutes a missed opportunity to investigate couple HIV sero-discordance and a failure of the intervention to reach the intended recipients.

The trial demonstrated that at least 2% of the couples were HIV sero-discordant. However, because of the low numbers of male partners tested, this figure is likely to be higher. Other studies in Uganda have reported rates of couples' HIV sero-discordance at 30% to 50% [23-26]. HIV sero-discordance is a key factor that influences rates of new infections among couples [36], thus increasing the risk of mother to child transmission of HIV during pregnancy, delivery and lactation.

The strength of this trial was the surprisingly comparable effect of a letter - a simple, cheap intervention that was easy to administer - in both arms. It could be argued that the main limitation of this trial was the high loss to follow up rate of approximately 40%, reducing the precision (internal validity) and the power of the trial to detect differences between the effect of the invitation letter and the information letter.

There are several possible reasons for the high rate of loss to follow up. Some pregnant women may have continued receiving antenatal care at lower level health units (health centres) nearest to their place of residence on learning from the midwives on their first antenatal clinic visit that they had low risk pregnancies. Others may not have attended follow-up ANC visits owing to transportation problems as the trial site was a referral hospital. Others could have attended clinics for HIV counselling and testing services and decided to continue with ANC elsewhere.

It is possible that community sensitization activities to encourage men to participate in ANC activities, as carried out in the Khayelitsha trial in South Africa [34], could have helped reduce the loss to follow up in our trial. Being a randomized, health facility-based trial, it is assumed that random allocation of the trial participants to the comparison groups, and masking of research assistants, health staff in the antenatal clinic and the participants, dealt with known and unknown confounders. As one of the health providers in the hospital, the principal investigator (RB) did not directly participate in administering intervention to the trial participants in order to avoid the Hawthorne effect on the internal validity of the trial. The findings of this trial could be generalized country-wide to populations that are similar to the one in the trial area.

## Conclusions

The effect of the intervention and the control on couple antenatal attendance was similar in both arms of the trial. In addition, this trial demonstrated that a simple intervention, such as a letter to the spouse, formulated as an invitation or as an information letter, could increase couple attendance by 10%. This intervention could be implemented in almost all African ANC clinics with PMTCT at a modest cost.

The trial also demonstrated that the majority (more than 90%) of the male partners who attended the antenatal clinic accepted HIV counselling and testing for HIV. Therefore, there is a requirement to evaluate this simple, cheap intervention further elsewhere in adequately powered studies to assess its effectiveness in increasing partner participation in antenatal clinics and the prevention of mother to child transmission of HIV. Such studies would better define the trial's implications for the PMTCT programme.

## Competing interests

The authors declare that they have no competing interests.

## Authors' contributions

RB participated in the conception, design and implementation of the trial, statistical analysis, interpretation of data and drafting of the manuscript. ANÅ participated in interpretation of data and the drafting of the manuscript. GN participated in the design of the trial, interpretation of data and drafting of the manuscript. CASK participated in interpretation of data and the drafting of the manuscript. TT participated in the conception and design of the trial, interpretation of data and drafting the manuscript. JKT participated in the design and implementation of the trial, interpretation of data and drafting of the manuscript. All authors read and approved the final manuscript.
